# Lead optimization for promising monoamine oxidase inhibitor from eugenol for the treatment of neurological disorder: synthesis and in silico based study

**DOI:** 10.1186/s13065-019-0552-4

**Published:** 2019-03-26

**Authors:** Priyanka Dhiman, Neelam Malik, Anurag Khatkar

**Affiliations:** Laboratory for Preservation Technology and Enzyme Inhibition Studies, Faculty of Pharmaceutical Sciences, M. D. University, Rohtak, Haryana 124001 India

**Keywords:** Neurological disorders, Monoamine oxidase, Eugenol derivatives, In silico design, Radical scavenging activity

## Abstract

Natural based inhibitors of monoamine oxidase are promising drug candidates for the treatment of several neurodegenerative and neuropsychological disorders including depression, anxiety, Parkinson’s disease and Alzheimer’s disease. In the present study we designed and synthesized the eugenol based derivatives and investigated them for human MAO inhibitory potential as promising candidates for therapeutics of neurological disorders. Moreover, radical scavenging activity of designed derivatives was tested by and H_2_O_2_ and DPPH scavenging methods. Eugenol based derivatives were designed and synthesized for human MAO inhibitory action. The in silico and in vitro models were utilized for the evaluation of hMAO inhibition. The insight into molecular interactions among the compounds and both hMAO-A and hMAO-B active site was achieved by molecular docking studies. The two spectrophotometric titrations techniques were used to evaluate antioxidant potential. Compounds **5b** and **16** were found as most active hMAO-A inhibitors with IC_50_ values of 5.989 ± 0.007 µM and 7.348 ± 0.027 µM respectively, through an appreciable selectivity index value of 0.19 and 0.14 respectively. In case of hMAO-B inhibition compounds **13a** and **13b** were found as most active hMAO-B inhibitors with IC_50_ values of 7.494 ± 0.014 µM and 9.183 ± 0.034 µM receptively and outstanding value of selectivity index of 5.14 and 5.72 respectively. Radical scavenging assay showed that compounds **5b**, **5a**, **9b**, **9a** were active antioxidants. The findings of present study indicated excellent correlation among dry lab and wet lab hMAO inhibitory experiments. Interestingly, the compounds exhibiting better MAO inhibition activity was also appeared as good antioxidant agents.

## Background

Nowadays major depressive disorders are affecting more than 12% population throughout the world in the form of stress and obsessive mood changes [1]. Around half of the all age group is enduring major depressive disorder (MDD) knowingly or unknowingly [2]. Most of the population with MDD generally not adequately responds to regularly approved drugs, for instance selective serotonin reuptake inhibitors (SSRIs). Moreover, millions of people worldwide are affected by neurodegenerative diseases [3]. Parkinson’s disease and Alzheimer’s disease are of the mainly widespread category, and among above five million Americans suffering with Alzheimer’s disease, and as a minimum 400,000 Americans suffering with Parkinson’s disease, though several approximations are much high [4]. Consequently, there is urgent need to develop effective anti-neurodegenerative and antidepressants drug based on natural products due to their lesser side effects. The inhibition of monoamine oxidase (MAO) is a significant way to treat neurodegenerative and depressive disorders.

**Fig. 1:**
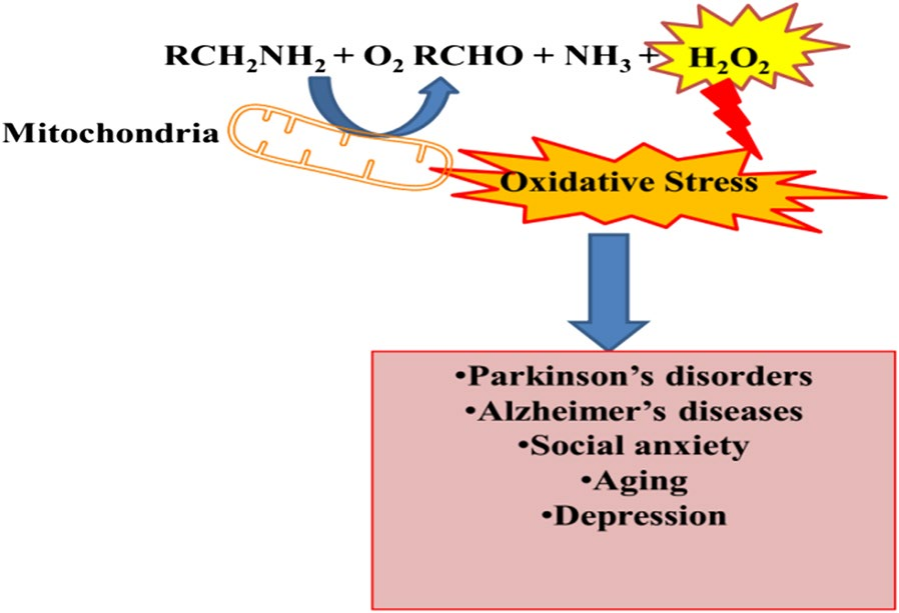
Graphical illustration of the monoaminergic neurotransmitter’s oxidative deamination to hydrogen peroxide and corresponding aldehyde

Monoamine oxidase (MAO; EC1.4.3.4) are mitochondrial membranous enzymes that catalyze the oxidative deamination of neurological catecholamines in the peripheral tissues and brain. The targeting of MAO is implicated in numerous mental disorders and renders psychological effects of some neurological drugs [5]. The metabolic by-products of reaction catalyze by MAO generates the corresponding aldehyde and ammonia along with hydrogen peroxide H_2_O_2_ which are highly neurotoxic [6] (Fig. 1). The overexpression of MAO or excess action by MAO initiates the neurodegenerative process, anxiety and depression. The resulted aldehyde molecule from deamination is associated with α-synuclein aggregation [7]. That leads to the Parkinson’s disease pathogenesis, however the H_2_O_2_ overproduction cause the oxidative damage and aggravates the apoptotic signaling actions. The MAO-A inhibition is associated to antidepressant activity, whereas inhibitors of MAO-B are related to the Parkinson’s disease treatment [8].

Moreover, the presently used MAO inhibitors are associated with numerous adverse effects, as hypertensive crises and liver toxicity [9]. Thus, the development of safe and effective MAO inhibitors is a challenge for the medicinal chemists to serve some potent and selective MAO inhibitors for the treatment of the neuropsychological and neurodegenerative disorders. The development of 3D crystallographic structure by Binda and coworkers has increased the enthusiasm of medicinal chemists to design more effective and targeted leads as MAO inhibitors [10]. By taking the advantage of modern computational methods such as molecular docking, absorption, distribution, metabolism, excretion and toxicity (ADMET), and QSAR along with synthetic medicinal chemistry might provide the target selective and effective MAO inhibitors.

**Fig. 2:**
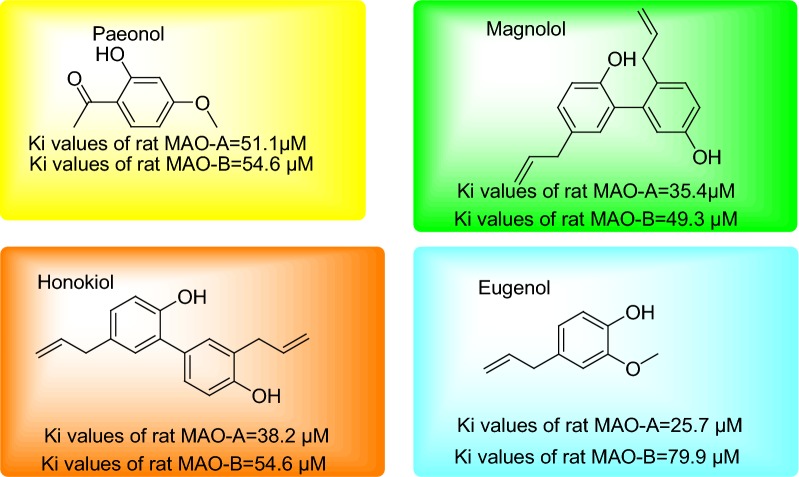
MAO inhibitory profile of eugenol related derivatives revealed in the current literature

Being the member of phenylpropanoids eugenol is chemically a substituted guaiacol via allyl chain. Eugenol is generally seeming as colorless to light yellow, oily liquid as aromatic essential oils extracted from basil, nutmeg, bay leaf, clove oil and cinnamon and its name is originated from *Eugenia caryophyllata* [11]. The general biosynthesis of eugenol occurs via amino acid tyrosine through sinapyl-alcohol dehydrogenase (SAD) forming coniferyl acetate and ultimately eugenol synthase to eugenol [12]. It is also worth to state the enormous work has been reported on natural phenols on MAO inhibition along with synthetic modifications and computational studies. Eugenol has attracted considerable attention because of its potential anticonvulsive, neuroprotective, anti-neurodegenerative activities and antidepressant. Moreover, many of these quoted the role of MAO for the neurological effects produced by eugenol [13]. Several reports also indicated the profound anti-oxidative effects of eugenol to reduce oxidative stress which is major cause of neurodegenerative disorders. Kong and coworkers evaluated the four phenols paeonol, honokiol, magnolol and eugenol for the MAO inhibitory potential from mitochondrial rat brain. Among all evaluated natural phenols eugenol showed significant MAO inhibitory potential [14] (Fig. 2).

Moreover, Clarke and coworkers evaluated eugenol from different biological sources as clove, oregano, nutmeg and cinnamon and administered to mice for 14 days. Eugenol significantly increased the forced swim test (FST) score. These observations show that eugenol cross the activity in a dose dependent manner blood–brain barrier in a small amount and act as a competitive human MAO-A inhibitor (Ki = 25 µM) and MAO B (Ki = 211 µM) [15]. Stafford et al. [16] further showed that the eugenol as a major active constituent within *Mentha aquatica* has remarkably inhibited MAO-B isoform by leaf extract within ethyl acetate at an IC_50_ value of 6 ± 5 μg/mL and within petroleum ether = 2 ± 1 μg/mL).

In another report by Klein-Júnior et al. [17] essential oils extracted from Eryngium species having eugenol as principle constituent showed notable monoamine oxidase inhibitory activity with IC_50_ value of 5.65 mg/mL. This study supported the fact that the natural species of Eryngium has eugenol as a chief bioactive secondary metabolite that potentially act on the central nervous system and could be promising drug agent for the treatment of neurodegenerative disorders. A comparative study conducted by Iriea and coworkers showed that eugenol display antidepressant-like activity via tail suspension test and forced swim test (FST) in mice better than imipramine (tricyclic antidepressant). The extracted eugenol from *Rhizoma acori graminei* stimulated hippocampus brain-derived neurotrophic factor (BDNF) and showed antidepressant-like activity which was not observed as with imipramine [18]. Similarly, Sousa and coworkers demonstrated the eugenol form *S. aromaticum* (L.) Merr. renders the antidepressant-like actions via inhibiting monoamine neurotransmission (MAO-A) at the lethal dose (LD50 = 4.5 g/kg) in mice. Moreover, repeated administration of eugenol decreased immobility in the FST and tail suspension test (TST) in a dose-dependent mode in mice [19] (Table 1).

**Table 1:** Natural phenols evaluated for MAO inhibition and animal behavioral studies

Natural phenols	Structure of MAO inhibitors	IC_50_ values of rat MAO-A (µM)	IC_50_ values of rat MAO-B (µM)	Animal specie	Behavioral test	Observations
Eugenol		5.34 ± 0.065	24.04 ± 1.32	Wistar rats	FST	Controls: negative and positive (ketamine)
Methyl-eugenol		7.12 ± 0.46	43.23 ± 0.054	Swiss albino rats (male and female)	FST	Controls: negative and positive (imipramine)
Menthol		23.11 ± 0.35	37.42 ± 0.76	ICR mouse	FST	Controls: negative and positive (fluoxetine)
Vanillin		17.88 ± 0.16	45.34 ± 0.09	Swiss mouse	FST	Controls: negative and positive (imipramine) The treatment reduced the spontaneous locomotion
Carvacrol		15.55 ± 0.49	23.42 ± 0.04	ICR mouse	FST	Controls: negative and positive (milnacipran)

Furthermore, some studies have postulated the mechanism of its neurological actions for e.g. Dubey et al. [20], explored the effect of eugenol on the amyloid formation of selected globular proteins. They revealed the neuroprotective nature of eugenol against toxic amyloids by stabilizing native proteins and to delay the conversion of protein species of native conformation into β-sheet assembled mature fibrils, which seems to be crucial for its inhibitory effect. Survey of compounds structurally related to eugenol has identified a few that inhibit MAOs more potently. Together, the activity of eugenol Mechan et al. [21] measured the MAO-A inhibition of oregano extract containing carvacrol. Both of the constituents exhibited selective MAO-A inhibitory activity in a dose dependent manner.

Tao and coworkers evaluated the eugenol from *Rhizoma acori graminei* for MAO inhibitory action and established the docking poses [22]. Eugenol showed inhibition constant for hMAO-A as Ki = 26 µM and Ki = 15 µM for hMAO-B. Docking simulation studies showed that eugenol interacted mainly with Tyr197, Tyr407, Asn181, Tyr444, Tyr69, and Gly443 active residues within the dynamic site. The aromatic phenolic ring was sandwiched between aromatic cases formed by Tyr444 and Tyr407 and this geometrical position lead to π–π stacking. Moreover, within the MAO-B active site, the aromatic phenolic ring found as embedded within Leu171 and Gln206 side chain.

**Fig. 3:**
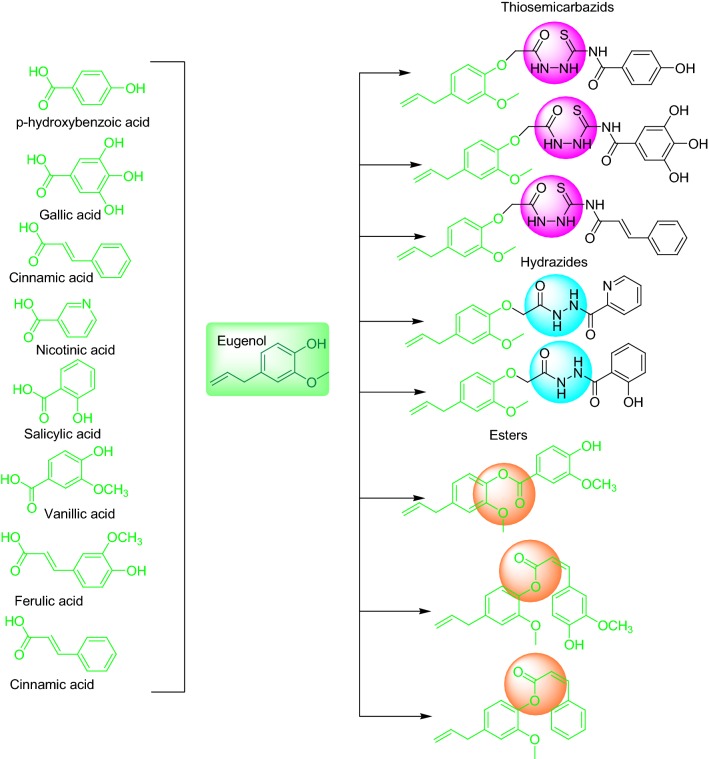
Design of strategy eugenol based derivatives for MAO inhibition and radical scavenging activity

Inspired by above-mentioned studies and their outcomes we designed and synthesized the eugenol based derivatives as MAO inhibitors (Fig. 3). Moreover, their radical scavenging activity was also evaluated to overcome oxidative stress. The molecular docking studies were performed to rationalize the selectivity for both MAO isoforms.

## Results and discussion

### Chemistry

**Scheme 1:**
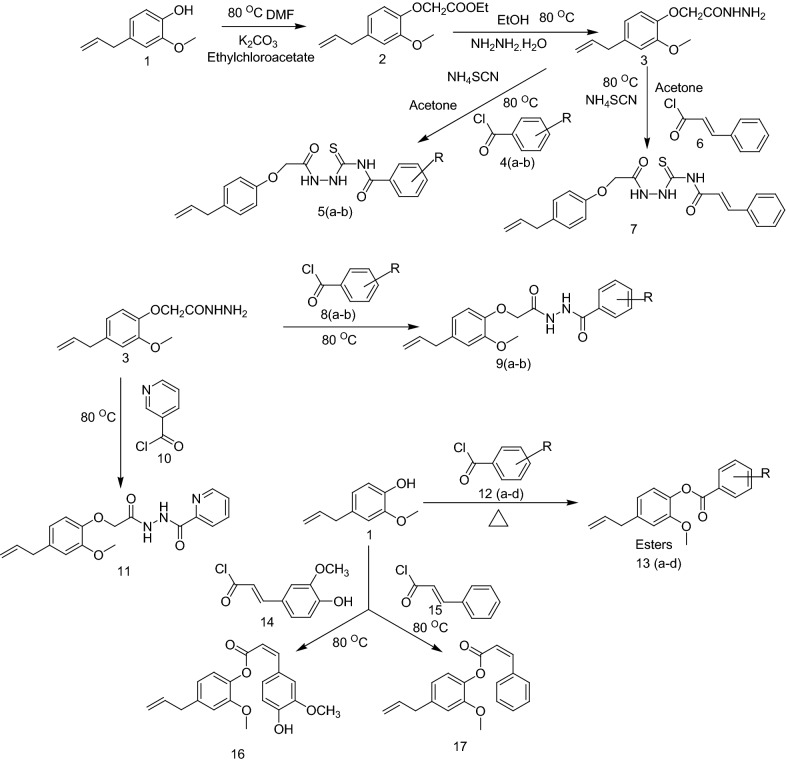
Schematic route followed for the synthesis of eugenol based derivatives

The key starting material, 2-(4-allyl-2-methoxyphenoxy)acetohydrazide (**3**) was prepared according to published procedures by Al-Amiery et al. [23]. The synthesis was initiated from eugenol (**1**) which is commercially available from Hi-media. The synthesis of ethyl 2-(4-allyl-2-methoxyphenoxy) acetate (**2**) was carried out by refluxing ethyl chloroacetate with eugenol in the presence of anhydrous K_2_CO_3_ in DMF at 80 °C for 24 h (Scheme 1). The confirmation of the formation of ethyl 2-(4-allyl-2-methoxyphenoxy)acetate (**2**) was evaluated by single spot TLC under UV lamp and IR as the phenolic 3400 cm^−1^ has been disappeared and the peak at 1735 cm^−1^ appeared to indicated formation of ester (Table 2). Moreover, the formation of ester was tested by ^1^HNMR indicating the disappearance of singlet 5.0 of phenolic OH. Finally, the 2-(4-allyl-2-methoxyphenoxy)acetohydrazide (3) was synthesized by refluxing ethyl 2-(4-allyl-2-methoxyphenoxy)acetate (**2**) with hydrazine hydrate in the presence of ethanol at 80 °C for 2–5 h. The IR spectrum of compound (**3**) confirmed a carbonyl group at 1739 cm^−1^, along with hydrazine amide group at 3268 cm^−1^. In the ^1^H-NMR; of compound (**3**) the allyl C–H (alkene) protons appeared at 6.43 ppm (*singlet*) and 5.83 ppm (*singlet*) and the *singlet* (NH) at 5.24 ppm.

**Table 2:** Physicochemical properties of synthesized compounds

Compound	Chemical structure	Mol. wt	Mol. formula	R_f_ value^a^	(%) Yield^a^	M.Pt (°C)
5a		385.44	C_19_H_19_N_3_O_4_S	0.67	75.4	234–235
5b		417.44	C_19_H_19_N_3_O_6_S	0.65	73.8	255–256
7		395.47	C_21_H_21_N_3_O_3_S	0.62	76.4	210–211
9a		356.37	C_19_H_20_N_2_O_5_	0.59	73.5	230–231
9b		372.44	C_19_H_20_N_2_O_4_S	0.74	72.8	210–211
11		341.36	C_18_H_19_N_3_O_4_	0.69	74.1	204–205
13a		314.33	C_18_H_18_O_5_	0.70	73.8	199–200
13b		284.31	C_17_H_16_O_4_	0.75	69.3	178–179
13c		284.31	C_17_H_16_O_4_	0.83	73.7	157–159
13d		300.37	C_17_H_16_O_3_S	0.84	75.9	182–184
16		340.37	C_20_H_20_O_5_	0.67	69.9	120–122
17		294.34	C_19_H_18_O_3_	0.79	72.4	178–179

^a^Mobile phase—**5a**–**11**-methanol-ethyl acetate–benzene (30:30:40 v/v/v), **13a**–**17**-acetone–toluene (30:70, v/v)

Finally, **5**(**a**–**b**) and compound **7** were prepared with compound (**3**) and different acid chlorides in the presence of ammonium thiocyanide by refluxing at 100 °C for 4–6 h (Table 3). The reaction completion was confirmed by TLC using the solvent systems methanol-ethyl acetate–benzene (30:30:40 v/v/v). And the single spots were visualized under UV lamp (255 and 360 nm). The ^1^H-NMR spectrum exhibited a *doublet* at 8.72–7.95 indicating the presence of –CO–NH–NH–SO– proton for e.g. compound **5a**. For compound **7**, the IR spectrum has the following characteristic absorption bands: 3377 (C=H str., Ar), 1614 (C=O str., 2^0^amide), 1177 (C–O–C assym. str., Ar), 1027 (C=S str., thiosemicarbazide), 945 (N–N str., N–NH). The ^13^CNMR peak at 178.9 indicated the presence of C=S bond formation in compound **7**. The ^1^H-NMR spectrum exhibited 7.65–7.59 multiplet showed the presence of secondary amide and at 3.51 doublet specified the amine group formation.

**Table 3:**
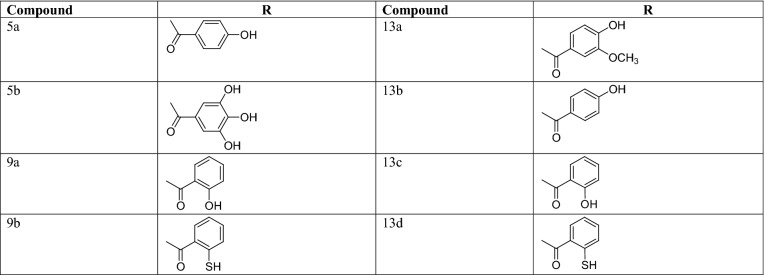
Substituent for the design of eugenol based derivatives (5a–13d)

The compounds **11** and **9**(**a**–**b**) were synthesized by (**3**) in the presence of nicotinoyl chloride and other natural based acid chlorides via reflux at 80 °C for 8 h in acetone. The IR peaks at 3440 indicated: phenolic O–H, 2960 cm^−1^ aromatic C=H, 1688 cm^−1^ secondary amide C=O, 1205 cm^−1^ aromatic C–O–C group, 1064 cm^−1^ thiosemicarbazide C=S, 917 cm^−1^ N–NH as N–N in compound **9a**. In the ^1^H-NMR spectrum doublet peaks at around 7.70 indicated the two secondary amide groups in compound **9a**. In case of compound **11** the IR peaks found as 3388 cm^−1^ for aromatic C=H bond, 1636 cm^−1^ N=CH for C=N group, 1614 cm^−1^ for secondary amide C=O, 1229 cm^−1^ for aromatic C–O–C group, 913 cm^−1^ N–NH for N–N groups. The ester derivatives **16**, **17**, **13**(**a**–**d**) were synthesized by refluxing eugenol with different natural based acid chlorides in the presence of pyridine at 80–90 °C for 8 h in diethyl ether. The confirmation for etherification reaction was done by single spot TLC using the solvent systems acetone–toluene (30:70, v/v). The IR spectra for compound **11** was found as 3077 cm^−1^ indicating aromatic C=H, 1700 cm^−1^ as ester formation as carbonyl group and 1206 cm^−1^ shown the aromatic C–O–C group. Moreover, the elemental analysis and mass spectrometric data were also found in agreement with compounds characterization.

### In vitro human MAO-A and human MAO-B inhibition

The results of the enzymatic experiments of the eugenol based derivatives compounds **5b** and **16** were found as most active hMAO-A inhibitors with IC_50_ values of 5.989 ± 0.007 µM and 7.348 ± 0.027 µM respectively, through an appreciable selectivity index value of 0.19 and 0.14 respectively (Table 4). 3,4,5-trihydroxybenzoy-thiosemicarbazide unit within the **5b** structure. Along with the location of carboxylic moiety in the 2-(4-allylphenoxy) acetyl nucleus the role of semicarbazides towards MAO inhibition has been observed. Moreover, the compound **16** exhibiting 4-hydroxy-3-methoxyphenyl-acrylate on 4-allyl-2-methoxyphenyl moiety has proven the importance of esteric linkage for h-MAOA active site. However, compound **17** with the absence of 2-methoxyphenyl groups at 3-phenylacrylate has appeared as a considerable hMAO-A inhibitor with an IC_50_ value 13.92 ± 0.051 µM and selectivity index of 0.40. Compound **7** showed significant hMAO-A inhibition with IC_50_ value 10.46 ± 0.072 µM and a selectivity index of 0.24. Presence of 4-cinnamoylthiosemicarbazide linked with 2-(4-allylphenoxy)acetyl ring in compound **7** shown that the compounds with acrylate group were more hMAO-A active than that of non acrylate unit. The reference compound chlorgyline showed hMAO-A inhibition with IC_50_ value 18.74 ± 0.096 µM. It has been reported that the active site of MAO-A is highly hydrophobic in nature which facilitates the large sized structures within it feasibly. So there has been a rational point for design the specific hMAO-A inhibitors with large aromatic structures.

**Table 4:** Human recombinant MAO inhibitory profile of eugenol based derivatives

Compound/sample	IC_50_ (µM)^a^ hMAO-A	IC_50_ (µM)^a^ hMAO-B	Selectivity index^b^	Docking score hMAO-A	Docking score hMAO-B
5a	15.85 ± 0.039	29.37 ± 0.048	0.53	− 9.65	− 3.23
5b	5.989 ± 0.007	31.19 ± 0.016	0.19	− 12.57	− 3.98
7	10.46 ± 0.072	43.31 ± 0.043	0.24	− 11.45	− 1.76
9a	10.84 ± 0.025	47.70 ± 0.073	0.22	− 11.34	− 1.01
9b	16.62 ± 0.004	27.85 ± 0.054	0.59	− 8.99	− 6.45
11	18.06 ± 0.044	26.80 ± 0.077	0.67	− 7.34	− 5.34
13a	38.53 ± 0.018	7.494 ± 0.014	5.14	− 2.45	− 11.87
13b	52.55 ± 0.038	9.183 ± 0.034	5.72	− 1.09	− 10.45
13c	22.38 ± 0.067	12.66 ± 0.021	1.76	− 6.45	− 10.32
13d	20.89 ± 0.023	13.95 ± 0.005	1.49	− 5.32	− 9.99
16	7.348 ± 0.027	49.15 ± 0.011	0.14	− 11.98	− 2.21
17	13.92 ± 0.051	34.20 ± 0.037	0.40	− 9.99	− 4.23
Eugenol	16.54 ± 0.045	13.30 ± 0.037	1.24	− 6.46	− 7.82
Pargyline	–	20.04 ± 0.095	–	− 6.061	–
Chlorgyline	18.74 ± 0.096	–	–	–	− 5.773

^a^Values related for the evaluated compound absorption which provide 50% inhibition of MAO-A and MAO-B, action, and are the mean SEM; statistical significance: p < 0.05 against the equivalent IC_50_ values achieved against MAO-A and MAO-B, as identified through ANOVA/Dunnett’s test

^b^Selectivity index = IC_50_ (MAO-A)/IC_50_ (MAO-B)

In the case of hMAO-B inhibition compounds **13a** and **13b** were found as most active hMAO-B inhibitors with IC_50_ values of 7.494 ± 0.014 µM and 9.183 ± 0.034 µM receptively and outstanding value of selectivity index of 5.14 and 5.72 respectively. Presence of 4-hydroxy-3-methoxybenzoate within the **13a** structure and small size of compound 13a contributed considerable hMAO-B inhibitory potential. In the case of compound **13b** the 4-hydroxybenzoate linkage with eugenol enhanced the hMAO-B inhibition. It was observed that methoxy group slightly reduced the hMAO-B inhibitory efficacy as compared with **13a** (having a single hydroxyl group at the aromatic ring). Moreover, the reference compound pargyline exhibited the value of 7.494 ± 0.014 µM for hMAO-B inhibition. As it is documented that hMAO-B enzyme composes of two cavities one is entrance cavity whereas another is substrate cavity, the amino acid residue Ile199 act as “gate keeper” for the substrate/inhibitor, so it depends upon the conformation of Ile199 (closed or open) for the interaction of the inhibitor compound. Moreover, the compounds **13a** and **13b** are smaller than other structures so they might be easily assessed the entrance cavity for inhibition.

### Enzyme kinetic for MAO inhibition

**Fig. 4:**
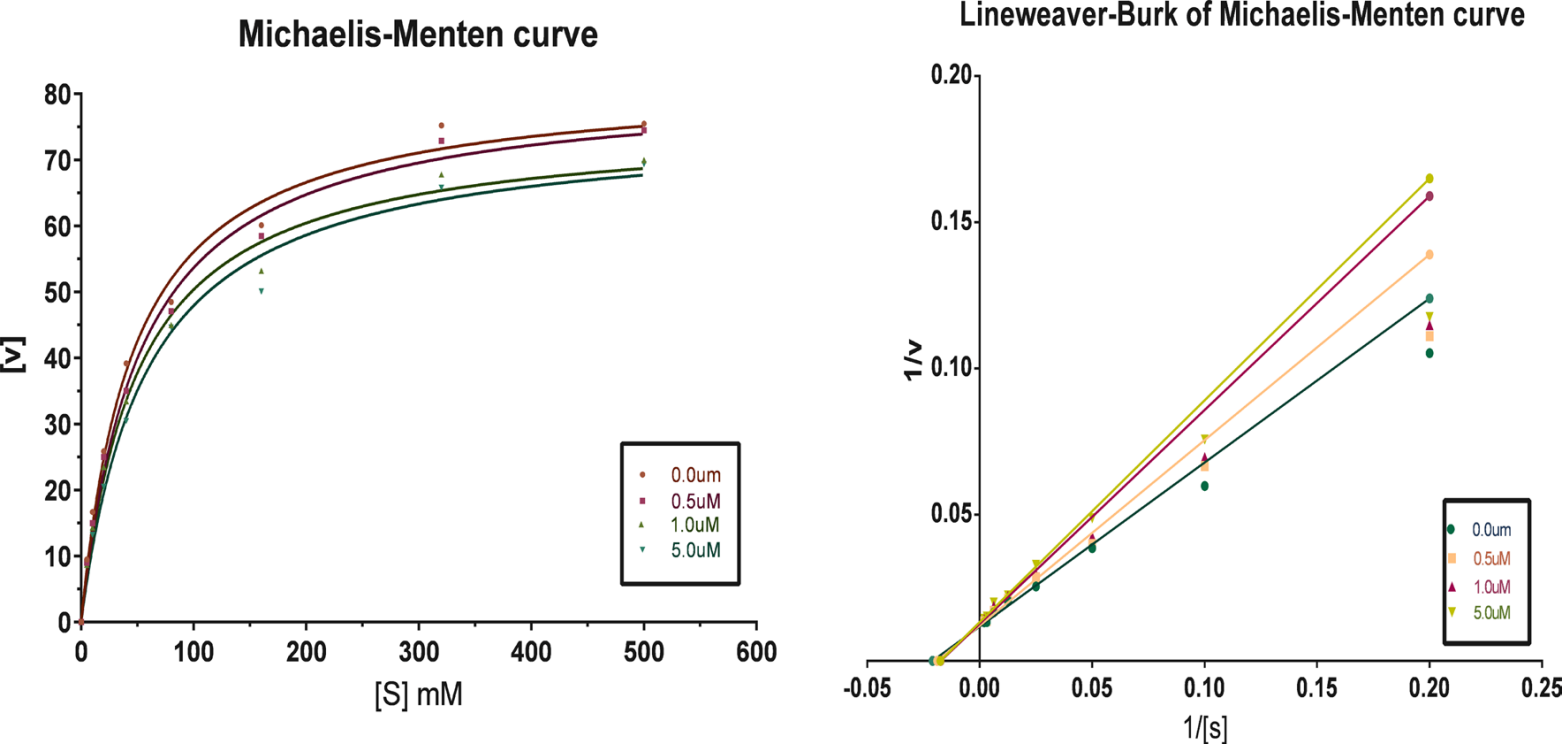
Enzyme kinetics observed for oxidation of tyramine by MAO-A in the absence and presence of various concentrations of the compound of **5b**

**Fig. 5:**
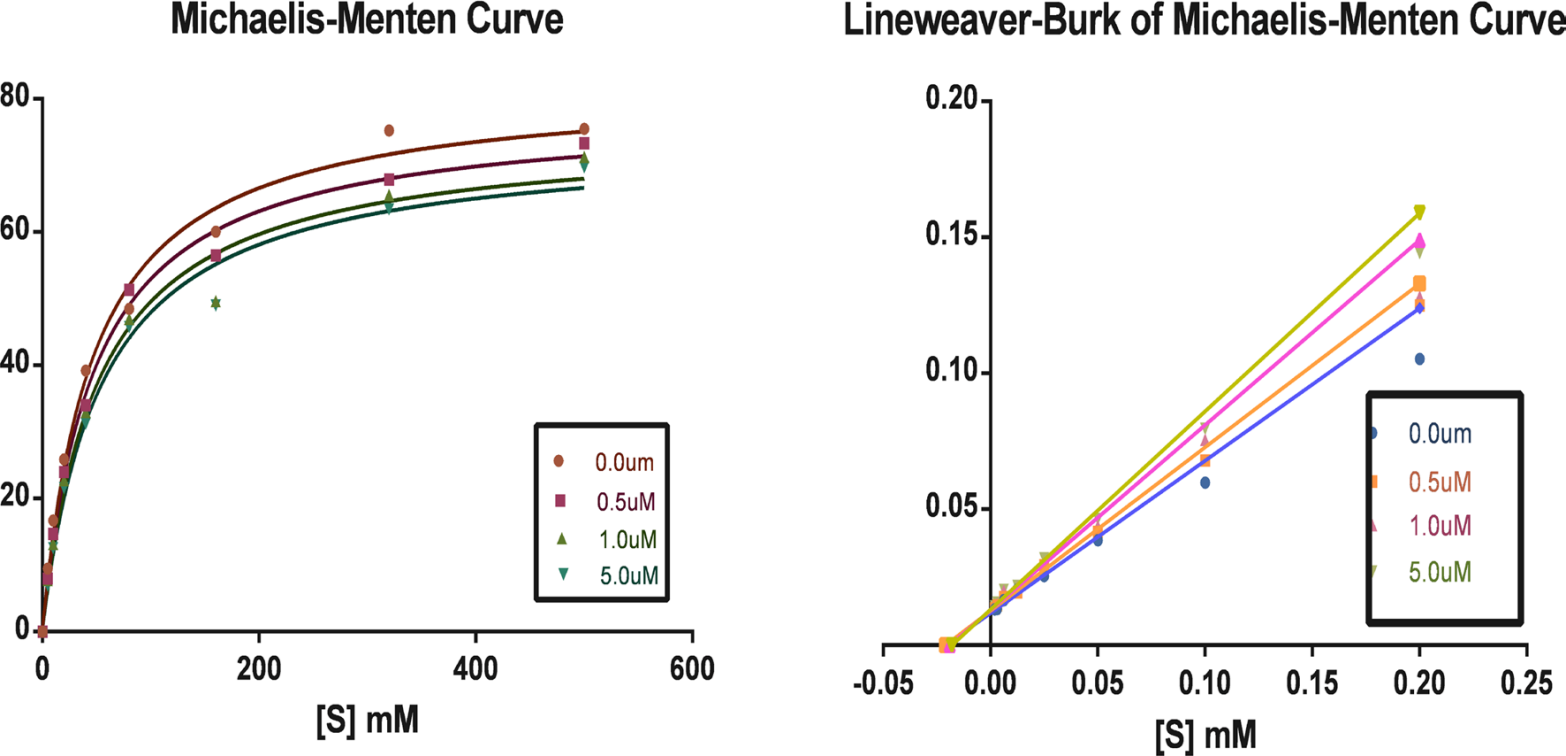
Enzyme kinetics observed for oxidation of tyramine by MAO-B in the absence and presence of various concentrations of the compound of **13a**

On the basis of the finding that compounds **5b** and **13a** are most potent hMAO-A and hMAO-B inhibitors, Lineweaver–Burk plots were constructed to understand the mechanism of inhibition MAO-A and MAO-B by these compounds. The Michaelis–Menten kinetics for both isozymes at their predetermined initial rates of reaction is presented as Lineweaver–Burk plot. Meanwhile, For each inhibitor, a set of six plots was constructed by employing eight different concentrations (15–250 μM) of the substrate (*p*-tyramine) for each plot. The inhibitor concentrations selected for the six plots were built. The intersection of all linear regression lines at y-axis and not x-axis indicates competitive mode of inhibition. These Lineweaver–Burk plots are shown in Figs. 4 and 5, and were indicative of a competitive inhibition of MAO-A by both compounds **5b** and **13a**.

### Statistical analysis

The outcomes of all studies are examined as mean ± SEM (standard error mean). Investigational results were calculated statistically by applying the analysis of one-way variance (ANOVA). Though, ANOVA showed significant variation as computed by ANOVA/Dunnett’s test. The *p* < 0.05 was observed as to be statistically considerable. The valuation of statistical parameters was performed by Graph Pad Prism 5.0 edition designed for Windows (San Diego, CA, USA).

### Docking studies

Molecular docking study was performed to insight the enzyme-inhibitor interactions for structural requirements for MAO activity and selectivity of eugenol based derivatives. Most active compound **5b** exhibited backbone hydrogen bonds Val93 via phenolic OH of 3,4,5-trihydroxybenzoyl unit. Moreover, one edge to face π–π stacking interaction (inter planer distance of ~ 5.04 Å) of 4-allyl-2-methoxyphenoxy and Phe112 was also appeared to support the ligand within the hMAO-A active site. The docking pose of compound **16** within the hMAO-A active site revealed a side chain hydrogen bond interaction with polar amino acid residue Asn181 through the hydroxyl group of the 4-hydroxy-3-methoxyphenyl-acrylate unit at inter planer distance of ~ 2.71 Å. Formation of an edge to face π–π stacking interaction between Phe108 and eugenol aromatic ring supported the ligand within the substrate cavity by inter planer distance of ~ 5.05 Å. Moreover, the ligand **16** was found as embedded within the active site through hydrophobic residues Cys323, Ala111, Val210, Phe108, Leu97, Ile180, and Tyr197 via eugenol aromatic ring. Some polar residues such as Gln215, Ser209, and Thr336 were found as engaged towards ester linkage of **16**. The 4-hydroxy-3-methoxyphenyl-acrylate unit was surrounded by an aromatic ring formed by Tyr407 and Tyr444, though the hydrophobic residues Leu337, Ile35, Tyr69, Phe352, and Tyr197 were also appeared near to compound **16**. Another hMAO-A active compound **9a** formed backbone hydrogen bonding with Val210 through oxygen of eugenol aromatic ring with inter planer distance of ~ 1.76 Å. The NH group of 2-hydroxybenzohydrazide formed another hydrogen bond with Phe208 via inter planer distance of ~ 2.23 Å. Moreover, the 2-hydroxybenzohydrazide unit of compound **9a** was supported by hydrophobic residues through Tyr407, Phe352, Met350, Ile180, Ile325, Leu337, Phe108, Cys323 and Ile335. Polar residues such as Gln215, Ser209, Asn212, Thr205, and Thr336 were attracted towards hydrazide linkage of compound **9a**.

**Fig. 6:**
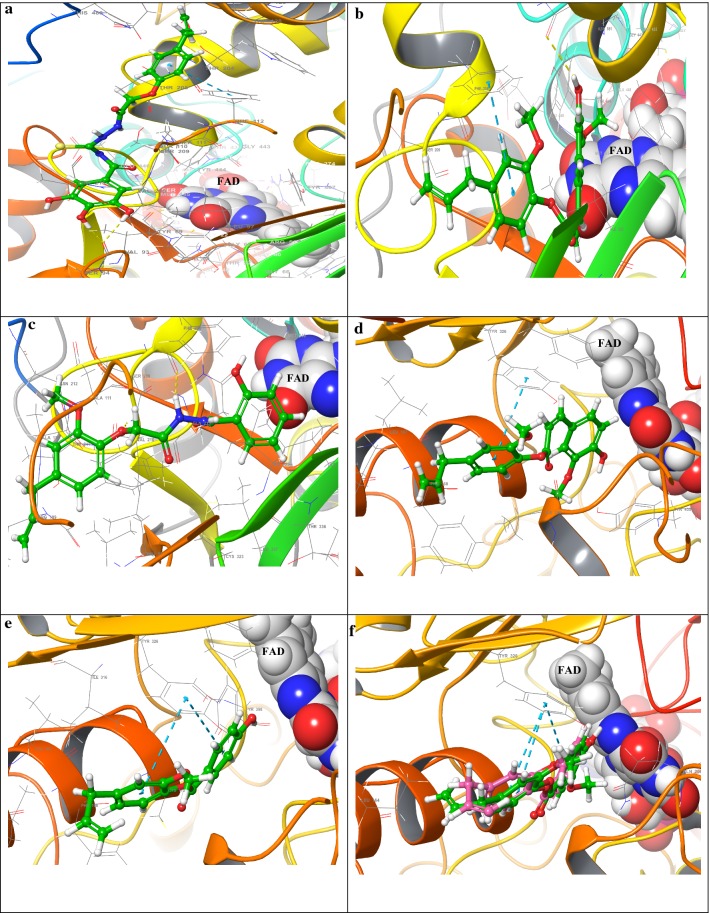
Docking poses of **5b**, **16**, **9a** within the hMAO-A active site in **a**–**c** whereas docking poses of **13a**, **13b** in **d**, and in **e** the superimposed pose of **13a**, **13b** within the hMAO-B active site. The dotted yellow lines indicate the hydrogen bonding whereas the blue lines indicate the π–π interactions

Docking pose of compound **7** showed an edge to face π–π stacking interaction between Phe108 and eugenol aromatic ring with inter-planer distance of ~ 5.34 Å. Interestingly, the eugenol unit was ideally accommodated within the active site by hydrophobic residues Cys323, Ile335, Met350, Phe352, Tyr69, Tyr407, Ile180, Ala111, Leu97 (Fig. 6).

In case of hMAO-B docking insight of compounds **13a** and **13b** showed two edges to face π–π stacking interaction between Tyr326 and both aromatic rings of compounds **13a** and **13b** through inter planer distance of ~ 5.24 Å and ~ 5.36 Å for 13a and ~ 5.29 Å and ~ 5.37 Å for 13b respectively. This outstanding behavior of **13a** and **13b** was noticed due to the small size of the compounds **13a** and **13b** as it has been reported that the active site of hMAO-B composes of two bipartite cavities. The inhibitor/substrate has to cross through two hydrophobic sub-cavities; the entrance cavity through dimensions of ~ 290 Å as well as the substrate cavity through dimensions of ~ 400 Å. Moreover both compounds **13a** and **13b** were surrounded by hydrophobic residues such as Phe168, Trp119, Leu167, Phe103, Pro104, Leu88, Pro102, Cys172, and Leu171. The aromatic case formed by residues Tyr188, Tyr398, Tyr435, Phe343; Tyr60 enclosed the 4-hydroxy-3-methoxybenzoate and 4-hydroxybenzoate units of **13a** and **13b** respectively. Most of the compound including eugenol showed hydrogen bonding with Pro102, and some of them interacted with gate keeper residue Ile199. The role of this gate keeper residue cannot be excluded from the discussion. The expedition of a substrate or inhibitor begin in the course of bendable loop (residue 99–112) which works as a gating residue at the outer mitochondrial membrane surface; the side chain isoleucine (Ile199) could exhibit two diverse conformations, closed conformation (separate both cavities) or open conformation (fuse both cavities) based on the character of the docked ligand. Maximum ligands showed edges to face π–π stacking with Trp119 and Tyr326.

### In silico ADMET profile of eugenol derivatives

The hypothetical evaluation of adsorption, distribution, metabolism, excretion and toxicity (ADMET) descriptors prediction was carried out by quick prop graphical user interface of Mastro-11.1 Schrödinger (Table 5) [24]. Evaluation of lipophilicity (logP) indicated the octanol–water partition coefficient that must be within limit 5. It could be predicted from the table that all of the eugenol based derivatives shown logP values within the limit. Lesser blood–brain barrier (BBB) access and poor membrane permeability is associated with a larger rate of tPSA (> 145 Å^2^) [32]. Nearly all of the synthesized derivatives exhibited acceptable values of tPSA within a range of 35–140 (Table 4). Based on Lipinski’s guide of 5 [25], below 5 hydrogen bond donors must be in lead, and the number of hydrogen bond acceptors (HBA) must be under 10. The aforesaid standard meets as well, because except **5b** (with 6 HBD) complete series of derivatives exhibited 0–4 HBD and 1–9 HBA. But this criterion is not fully reliable and has been challenged in case of natural potent well known therapeutic agents. All of the derivatives could be considered to exhibits fine oral bioavailability, examined as confirmed by Veber rule  [26]. This states that the rotatable bonds magnitude should be correspondent to 10 and tPSA ≤ 140 Å, or else the whole no. of hydrogen bond donor and acceptor (HBA + HBD) must be ≤ 12. It is considerable that not any of derivatives was discarded, and each one of the derivatives proved promising ADMET rank (Table 4).

**Table 5:** In silico ADMET profile of eugenol derivatives

Compound/sample	Mol. Wt	TPSA	No. of rotatable bonds	DonorHB	AccptHB	QPlogPo/w	QPlogBB	QPPMDCK	QPPCaco
5a	385.44	99.69	9	4	7	2.43	0.234	2234.34	3124.44
5b	417.44	140.14	9	6	9	1.65	0.245	3345.35	2567.54
7	395.47	79.46	10	3	6	3.55	0.755	2575.35	3211.08
9a	356.37	96.89	8	3	7	2.79	0.145	3566.34	4237.75
9b	372.44	76.66	8	2	6	2.49	0.467	523.133	345.723
11	341.36	89.56	8	2	7	1.14	0.543	346.234	978.233
13a	314.33	65.00	7	1	5	3.56	1.245	393.234	967.654
13b	284.31	55.77	6	4	1	3.74	1.456	267.912	745.234
13c	284.31	55.77	6	1	4	4.24	0.387	2724.23	4120.67
13d	300.37	35.54	6	0	3	4.06	0.233	4452.45	3243.34
16	340.37	65.00	8	1	5	3.38	0.234	1234.23	2345.07
17	294.34	35.54	7	0	3	4.04	0.532	2233.12	3886.12

### Determination of DPPH scavenging activity

**Fig. 7:**
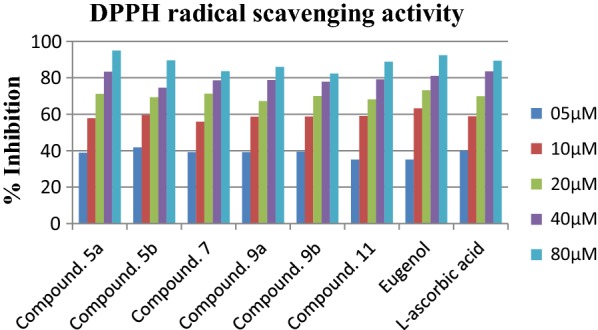
DPPH radical scavenging activity of most active compounds with respect to reference l-ascorbic acid

Results of DPPH scavenging assay showed that compounds **5b**, **5a**, **9b**, **9a** were most active antioxidants with IC_50_ values of 8.304 ± 0.073 µM, 9.152 ± 0.063 µM, 9.293 ± 0.028 µM, 9.514 ± 0.017 µM respectively (Table 6). It was observed that these compounds were possessing a large number of hydrogen donors as NH and OH groups within their structures. Presence of these groups generally considered to be contributors for potential antioxidant effect by polyphenolic compounds. However, the eugenol and reference compound l-ascorbic acid exhibited the IC_50_ value of 10.29 ± 0.011 and 8.58 ± 0.009 respectively. Other compounds such as compounds **7** and **11** were also appeared to be considerable antioxidants with IC_50_ values of 9.786 ± 0.012 µM and 11.21 ± 0.044 µM respectively (Fig. 7). It is notable that the compound **7** was observed as a poor agent for DPPH scavenging, the reason for this might be the absence of any OH or NH group with the compound **7** structure.

**Table 6:** Determination of DPPH scavenging activity of eugenol derivatives

Compound/sample	IC_50_ (µg/mL)^a^	Sr. no	IC_50_ (µg/mL)^a^
5a	9.152 ± 0.063	13a	20.66 ± 0.032
5b	8.304 ± 0.073	13b	12.47 ± 0.014
7	9.786 ± 0.012	13c	16.04 ± 0.026
9a	9.514 ± 0.017	13d	14.88 ± 0.018
9b	9.293 ± 0.028	16	27.70 ± 0.023
11	11.21 ± 0.044	17	31.30 ± 0.014
Eugenol	10.29 ± 0.011	l-Ascorbic acid	8.58 ± 0.009

^a^Value are expressed as mean ± SEM, n = 3

### H_2_O_2_ scavenging activity

**Fig. 8:**
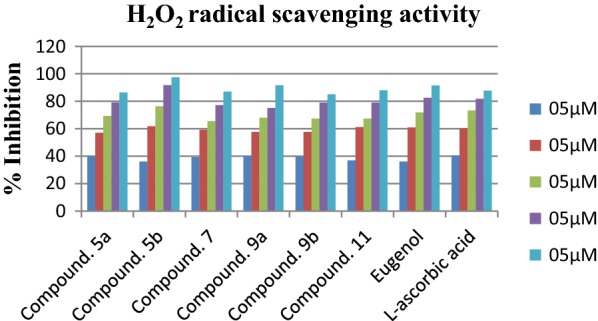
Hydrogen peroxide scavenging (H_2_O_2_) activity of most active compounds with respect to reference l-ascorbic acid

The H_2_O_2_ scavenging assay was carried out for further evaluate the antioxidant potential of synthesized compounds. In cases of H_2_O_2_ scavenging the compounds **5b**, **5a**, **9b**, **9a** were most active H_2_O_2_ scavengers with IC_50_ values of 8.904 ± 0.034 µM, 9.156 ± 0.002 µM, 9.393 ± 0.017 µM, 9.547 ± 0.051 µM respectively (Table 7). Surprisingly, the compounds with good DPPH scavenging profile were observed as considerable H_2_O_2_ scavengers. Thus, the presence of the OH and NH groups is essential for the antioxidant activity. However, eugenol and reference compound l-ascorbic acid exhibited the IC_50_ values of 9.980 ± 0.012 µM and 8.121 ± 0.082 µM respectively (Fig. 8). Compounds **7** and **11** were showing similar behavior towards H_2_O_2_ and the IC_50_ values of them were 9.755 ± 0.021 µM and 10.33 ± 0.022 µM respectively.

**Table 7:** H_2_O_2_ scavenging activity of eugenol derivatives

Compound/sample	IC_50_ (µg/mL)^a^	Sr. no	IC_50_ (µg/mL)^a^
5a	9.156 ± 0.002	13a	26.6 ± 0.544
5b	8.904 ± 0.034	13b	12.5 ± 0.862
7	9.755 ± 0.021	13c	15.7 ± 0.784
9a	9.547 ± 0.051	13d	14.4 ± 0.143
9b	9.393 ± 0.017	16	20.6 ± 0.856
11	10.33 ± 0.022	17	30.4 ± 0.064
Eugenol	9.980 ± 0.012	l-Ascorbic acid	8.121 ± 0.082

^a^Value are expressed as mean ± SEM, n = 3

### Nitric oxide scavenging activity

**Fig. 9:**
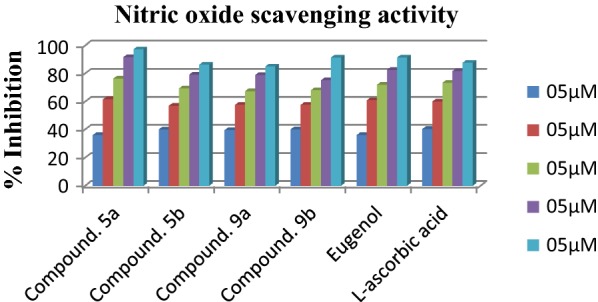
Nitric oxide scavenging activity of most active compounds with respect to reference L-ascorbic acid

To validate the results of above mentioned antioxidant activity assays we carried out the nitric oxide scavenging activity of the titled compounds. Results of nitric oxide scavenging activity showed that the compound **5b** was found as most active NO· scavenger with IC_50_ value of 6.456 ± 0.023 µM as compared with eugenol (IC_50_ value 11.724 ± 0.003 µM) and reference compound l-ascorbic acid with IC_50_ values of 7.523 ± 0.005 µM (Table 8). Moreover compounds **5a**, **9b**, **9a** were also observed as mild active NO· scavengers with IC_50_ values of 7.156 ± 0.002 µM, 8.095 ± 0.018 µM, 9.007 ± 0.014 µM respectively (Fig. 9). The result of the nitric oxide scavenging activity showed similar routine for the antioxidant action as observed with DPPH and H_2_O_2_ scavenging profile.

**Table 8:** Nitric oxide scavenging activity of eugenol derivatives

Compound/sample	IC_50_ (µg/mL)^a^	Sr. no	IC_50_ (µg/mL)^a^
5a	7.156 ± 0.002	13a	20.23 ± 0.073
5b	6.456 ± 0.023	13b	11.74 ± 0.523
7	9.142 ± 0.061	13c	14.63 ± 0.412
9a	9.007 ± 0.014	13d	11.23 ± 0.032
9b	8.095 ± 0.018	16	18.63 ± 0.654
11	10.71 ± 0.743	17	25.61 ± 0.076
Eugenol	11.724 ± 0.003	l-Ascorbic acid	7.523 ± 0.005

^a^Value are expressed as mean ± SEM, n = 3

### Ferric reducing antioxidant power (FRAP) assay

**Fig. 10:**
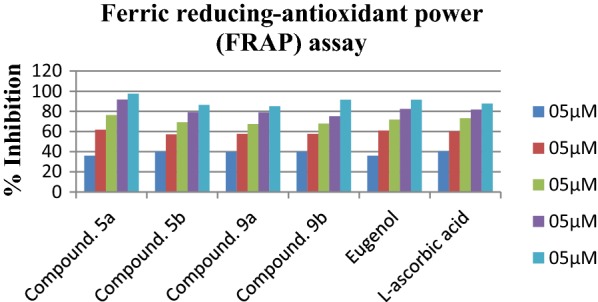
Ferric reducing antioxidant power (FRAP) of most active compounds with respect to reference l-ascorbic acid

Assay of ferric reducing antioxidant power (FRAP) assay showed that compound was compound **5b** found as most active antioxidant with IC_50_ value of 5.112 ± 0.009 µM as compared with eugenol (IC_50_ value 10.627 ± 0.082 µM) and reference compound l-ascorbic acid with IC_50_ values of 8.112 ± 0.091 µM (Table 9). However compounds **5a**, **9a**, **9b** were also observed as considerable antioxidant agents with IC_50_ value of 5.934 ± 0.053 µM, 7.234 ± 0.001 µM, 9.912 ± 0.005 µM respectively (Fig. 10).

**Table 9:** Ferric reducing antioxidant power (FRAP) of eugenol derivatives

Compound/sample	IC_50_ (µg/mL)^a^	Sr. no	IC_50_ (µg/mL)^a^
5a	5.934 ± 0.053	13a	18.14 ± 0.001
5b	5.112 ± 0.009	13b	10.77 ± 0.026
7	9.142 ± 0.061	13c	14.63 ± 0.007
9a	7.234 ± 0.001	13d	12.45 ± 0.017
9b	9.912 ± 0.005	16	16.88 ± 0.099
11	9.126 ± 0.013	17	15.91 ± 0.003
Eugenol	10.627 ± 0.082	l-Ascorbic acid	8.112 ± 0.091

^a^Value are expressed as mean ± SEM, n = 3

### Structure–activity relationship (SAR) studies

**Fig. 11:**
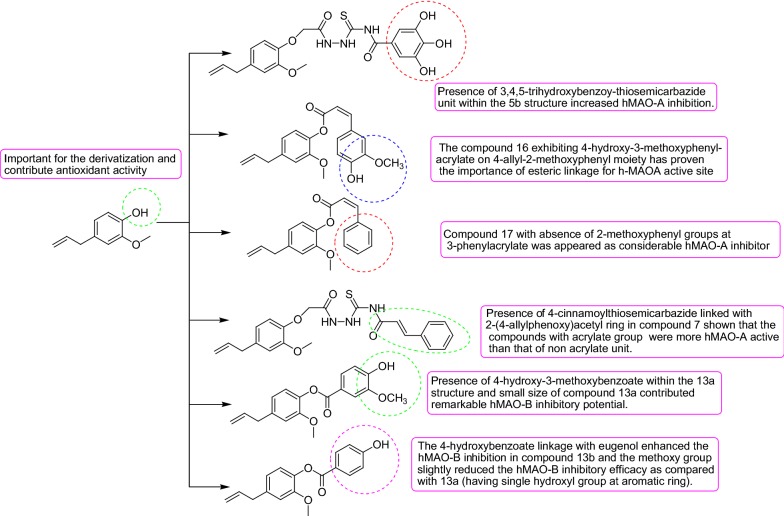
Through insight into an examination of the antioxidant MAO screening assay explicated of some SARs

The structure–activity relationship of the evaluated eugenol based derivatives by means of their antioxidant activity and MAO inhibitory activity and results is summarized in Fig. 11.

## Experimental

### Materials and methods

Unless otherwise noted, the analytical grade chemicals required for synthesis and antioxidant activity procured from Hi-media Laboratories. The biological evaluation of the test drugs on hMAO activity was investigated by measuring their effects on the production of hydrogen peroxide (H_2_O_2_) from p-tyramine (a common substrate for hMAO-A and hMAO-B), using the Amplex Red MAO assay kit (Sigma USA) and microsomal MAO isoforms prepared from insect cells (BTI-TN-5B14) infected with recombinant baculovirus containing cDNA inserts for hMAO-A or hMAO-B (Sigma-Aldrich USA) were used as source for the two microsomal MAO isoforms. Reactions were monitored by thin layer chromatography TLC performed on 0.25 mm precoated silica gel plates procured from Merck, and the spots were visualized in iodine chamber and UV, in mobile media TLC-(**5a–11)**-methanol-ethyl acetate–benzene (30:30:40 v/v/v), (**13a**–**17**)-acetone–toluene (30:70, v/v). Melting points were determined in open capillary tubes on a Sonar melting point apparatus. The nuclear magnetic resonance (NMR) spectra 1H NMR and 13C NMR spectra were recorded in DMSO and deuterated CDCl_3_ respectively on spectrometer (Model: Bruker Avance II 400 NMR) at a frequency of 400 MHz downfield to tetramethylsilane standard and chemical shifts were recorded as d (parts per million) and s, d, t, q, m, dd, td, dt and tt are designated as singlet, doublet, triplet, quartet, multiplet, doublet of doublets, triplet of doublets, doublet of triplets and triplet of triplets respectively. Coupling constants (J) were reported in hertz (Hz). Infrared (IR) spectra were recorded on Perkin Elmer FTIR spectrophotometer by using KBr pellets technique.

### General procedure for the synthesis of acid chlorides

A mixture of different aromatic carboxylic acids and thionyl chloride (10 mL) was stirred and refluxed 80 °C for 2–4 h in the presence of pyridine (1–2 drops) till the completion of reaction. Distillation of the reaction mixture removed the excess of thionyl chloride. Finally the purity of resulting product was appropriate to use it directly for the following synthesis.

### General procedure for preparation of eugenol esters (**13a**–**17**)

The target esters derivatives derived from eugenol, which were prepared by gently refluxing a solution of eugenol in ether (50 mL) with a solution of different aromatic acid chlorides (0.05 mol) in diethyl ether (50 mL).

The reaction mixture was heated on a water bath (80 °C) until the complete removal of hydrogen chloride and completion of reaction was checked by single spot TLC. The shiny white precipitates so obtained were filtered using suction and rendered free from chloride by repeated washing with water and finally purified by recrystallization with acetone.

### General procedure for preparation of 2-(4-allyl-2-methoxyphenoxy)acetohydrazide

Synthesis of 2-(4-allyl-2-methoxyphenoxy)acetohydrazide (0.01 mol) was brought about by refluxing (80 °C) ethyl bromoacetate (0.01 mol) with eugenol (0.01 mol) in the presence of anhydrous K_2_CO_3_ in dry DMF for 24 h. Which yielded compound ethyl 2-(4-allyl-2-methoxyphenoxy)acetate, further reaction was followed by the addition of hydrazine hydrate (0.014 mol) to yield 2-(4-allyl-2-methoxyphenoxy)acetohydrazide by refluxing 2–5 h at 80 °C. The crude solid was filtered, dried and recrystallised with mixture of chloroform–methanol (1:2).

### General procedure for preparation of 1-(2-(4-allylphenoxy)acetyl)-4phenyl-thiosemicarbazides

5(a–b) and compound 7 were synthesized by refluxing (80 °C) the 2-(4-allyl-2-methoxyphenoxy)acetohydrazide (0.01 mol) with the different aromatic acid chlorides (0.01 mol) and cinnamyl chloride (0.01 mol) in the presence of ammonium thiocyanate and acetone for 8 h. Following recrystallisation from ethanol, the target 1-(2-(4-allylphenoxy)acetyl)-4phenyl-thiosemicarbazides were obtained in appreciable yields.

### General procedure for preparation of *N*′-(2-(4-allyl-2-methoxyphenoxy)acetyl)benzohydrazides and *N*′-(2-(4-allyl-2-methoxyphenoxy)acetyl)picolinohydrazide

Target compound 2-(4-allyl-2-methoxyphenoxy)acetohydrazides (0.01 mol) were prepared in relatively high yields by reacting the appropriately different aromatic carboxylic acids (0.01 mol) and nicotinoyl chloride (0.01 mol) on reflux (80 °C) for 8 h in acetone. After reaction completion, the solid product was washed with ether and recrystallization was done with ethanol to yield corresponding *N*′-(2-(4-allyl-2 methoxyphenoxy)acetyl)benzohydrazides and *N*′-(2-(4-allyl-2 methoxyphenoxy)acetyl)picolinohydrazide.

### Spectral data

**5a** 1-(2-(4-allylphenoxy)acetyl)-4-(4-hydroxybenzoyl)thiosemicarbazide IR (KBr pellets) cm^−1^ 3359 (O–H str., phenol), 3070 (C=H str., Ar), 1663 (C=N str., N=CH), 1602 (C=O str., 2^0^ amide), 1273 (C–O–C assym. str., Ar), 1174 (C=S str., thiosemicarbazide), 943 (N–N str., N–NH). ^1^H NMR (400 MHz, DMSO-*d*_6_): 8.72–7.95 (m, 2H), 7.05 (ddd, *J* = 7.5, 2.2, 1.1 Hz, 4H), 6.99–6.82 (m, 2H), 5.78 (ddt, *J* = 16.8, 9.9, 6.2 Hz, 1H), 5.19–5.08 (m, 2H), 4.68 (s, 2H), 3.48 (dp, *J* = 6.2, 1.0 Hz, 2H). ^13^C NMR (400 MHz, CDCl_3_): 180.5, 165.6, 163.7, 159.8, 155.2, 136.9, 135.1, 127.9, 127.1, 126.8, 113.9, 113.4 (d, *J* = 8.8 Hz), 66.9, 45.6; m/z 415.09 [M+1]+; Anal. For C_20_H_21_N_3_O_5_S: calcd: C, 57.82; H, 5.09; N, 10.11, found: C, 57.80; H, 5.07; N, 10.10.

**5b** 1-(2-(4-allylphenoxy)acetyl)-4-(3,4,5-trihydroxybenzoyl)thiosemicarbazide IR (KBr pellets) cm^−1^ 3407 (O–H str., phenol), 3286 (C=H str., Ar), 1705 (C=N str., N=CH), 1626 (C=O str., 2^0^ amide), 1177 (C–O–C assym. str., Ar), 1030 (C=S str., thiosemicarbazide), 944 (N–N str., N–NH). ^1^H NMR (400 MHz, DMSO-*d*_6_): 7.11 (d, *J* = 7.2 Hz, 4H), 6.80–6.72 (m, 2H), 5.99 (tt, *J* = 13.4, 6.2 Hz, 1H), 5.31 (dddt, *J* = 21.1, 13.4, 2.1, 1.0 Hz, 2H), 4.68 (s, 2H), 3.45 (dp, *J* = 6.2, 1.0 Hz, 2H). ^13^C NMR (400 MHz, CDCl_3_): 191.5, 174.6, 175.8, 159.2, 147.6, 138.7, 137.9, 134.1, 129.1, 128.3, 115.4 (d, *J* = 8.8 Hz), 104.7, 65.9, 39.6; m/z 447.12 [M+1]+; Anal. For C_20_H_21_N_3_O_7_S: calcd: C, 53.68; H, 4.73; N, 9.39, found: C, 53.67; H, 4.72; N, 9.36.

**7** (*E*)-1-(2-(4-allylphenoxy)acetyl)-4-cinnamoylthiosemicarbazide IR (KBr pellets) cm^−1^ 3377 (C=H str., Ar), 1614 (C=O str., 2^0^ amide), 1177 (C–O–C assym. str., Ar), 1027 (C = S str., thiosemicarbazide), 945 (N–N str., N–NH). ^1^H NMR (400 MHz, DMSO-*d*_6_): 7.65–7.59 (m, 4H), 7.52 (dt, *J* = 15.1, 0.6 Hz, 2H), 7.49–7.37 (m, 4H), 7.33–7.31 (m, 2H), 7.07–6.88 (m, 4H), 6.86–6.80 (m, 4H), 6.76 (d, *J* = 15.1 Hz, 2H), 5.94 (ddt, *J* = 16.8, 9.9, 6.2 Hz, 2H), 5.18–5.11 (m, 3H), 5.07 (dt, *J* = 2.1, 1.0 Hz, 1H), 4.56 (s, 4H), 3.51 (dp, *J* = 6.2, 1.0 Hz, 4H). ^13^C NMR (400 MHz, CDCl_3_): 178.9, 169.6, 163.8, 157.2, 140.9, 138.9, 135.0 (d, *J* = 17.5 Hz), 131.1, 129.0 (d, *J* = 18.3 Hz), 127.1, 122.3, 117.4 (d, *J* = 8.8 Hz), 70.9, 39.6; m/z 447.12 [M+1]+; Anal. For C_22_H_23_N_3_O_4_S: calcd: C, 62.10; H, 5.45; N, 9.88, found: C, 62.09; H, 5.44; N, 9.87.

**9a** *N*′-(2-(4-allyl-2-methoxyphenoxy)acetyl)-2-hydroxybenzohydrazide IR (KBr pellets) cm^−1^ 3440 (O–H str., phenol), 2960 (C=H str., Ar), 1688 (C=O str., 2^0^ amide), 1205 (C–O–C assym. str., Ar), 1064 (C=S str., thiosemicarbazide), 917 (N–N str., N–NH). ^1^H NMR (400 MHz, DMSO-*d*_6_): 7.70 (dd, *J*=7.4, 1.5 Hz, 2H), 7.43 (td, *J* = 7.5, 1.5 Hz, 2H), 7.01–6.93 (m, 4H), 6.88 (dt, *J* = 7.7, 0.8 Hz, 2H), 6.85 (dp, *J* = 7.8, 1.0 Hz, 4H), 5.55 (dd, *J* = 16.8, 9.9, Hz, 2H), 5.15–5.09 (m, 3H), 5.03 (dt, *J* = 2.2, 1.0 Hz, 1H), 4.77 (s, 4H), 3.38 (s, 6H), 3.31 (dp, *J* = 6.2, 1.0 Hz, 4H). ^13^C NMR (400 MHz, CDCl_3_): 170.7, 167.7, 157.7, 152.8, 151.1, 145.2, 144.1, 143.2, 129.4, 124.2, 117.4, 115.5 (d, *J* = 5.5 Hz), 113.5, 113.2, 111.3, 67.8, 57.7, 41.4; m/z 356.12 [M+1]+; Anal. For C_19_H_20_N_2_O_5_: calcd: C, 64.04; H, 5.66; N, 7.86, found: C, 64.03; H, 5.65; N, 7.85.

**9b** *N*′-(2-(4-allyl-2-methoxyphenoxy)acetyl)-2-mercaptobenzohydrazide IR (KBr pellets) cm^−1^ 3378 (O–H str., phenol), 3094 (C=H str., Ar), 1716 (C=O str., 2^0^ amide), 1260 (C–O–C assym. str., Ar), 917 (N–N str., N–NH), 697 (C=S str., thiol). ^1^H NMR (400 MHz, DMSO-*d*_6_): 7.72 (dd, *J* = 7.4, 1.5 Hz, 2H), 7.48 (dd, *J* = 7.4, 1.6 Hz, 2H), 7.38 (td, *J* = 7.5, 1.5 Hz, 2H), 7.25 (td, *J* = 7.4, 1.6 Hz, 2H), 6.88 (dt, *J* = 7.7, 0.8 Hz, 2H), 6.75 (dp, *J* = 7.8, 1.0 Hz, 4H), 5.96 (ddt, *J* = 16.8, 9.9, 6.2 Hz, 2H), 5.15–5.13 (m, 3H), 5.10 (dt, *J* = 2.2, 1.0 Hz, 1H), 4.73 (s, 4H), 3.87 (s, 6H), 3.35 (dp, *J* = 6.2, 1.0 Hz, 4H). ^13^C NMR (400 MHz, CDCl_3_): 169.7, 165.2, 150.8, 146.1, 139.2, 137.1, 135.8, 132.5, 130.8, 128.4 (d, *J* = 16.0 Hz), 127.5, 120.2, 115.3, 113.5, 110.3, 67.8, 55.7, 40.4; m/z 372.10 [M+1]+; Anal. For C_19_H_20_N_2_O_4_S: calcd: C, 61.27; H, 5.41; N, 7.52, found: C, 61.25; H, 5.40; N, 7.51.

**11** *N*′-(2-(4-allyl-2-methoxyphenoxy)acetyl)picolinohydrazide IR (KBr pellets) cm^−1^ 3388 (C = H str., Ar), 1636 (C=N str., N=CH), 1614 (C=O str., 2^0^ amide), 1229 (C–O–C assym. str., Ar), 913 (N–N str., N–NH). ^1^H NMR (400 MHz, DMSO-*d*_6_): 8.76–8.69 (m, 2H), 8.10–8.00 (m, 4H), 7.81 (ddd, *J* = 7.5, 5.9, 3.1 Hz, 2H), 6.89 (dt, *J* = 7.7, 0.9 Hz, 2H), 6.77 (dp, *J* = 7.8, 1.0 Hz, 4H), 5.99 (ddt, *J* = 16.8, 9.9, 6.2 Hz, 2H), 5.18–5.10 (m, 3H), 5.09 (dt, *J* = 2.2, 1.0 Hz, 1H), 4.79 (s, 4H), 3.85 (s, 6H), 3.30 (dp, *J* = 6.2, 1.0 Hz, 4H). ^13^C NMR (400 MHz, CDCl_3_): 170.7, 166.7, 151.6, 150.8, 150.6, 146.1, 141.2, 140.9, 140.1, 125.1, 123.1 (d, *J* = 16.0 Hz), 117.3, 115.5, 112.3, 79.8, 55.7, 40.4; m/z 341.11 [M+1]+; Anal. For C_18_H_19_N_3_O_4_: calcd: C, 63.33; H, 5.61; N, 12.31, found: C, 63.30; H, 5.59; N, 12.30.

**13a** 4-allyl-2-methoxyphenyl 4-hydroxy-3-methoxybenzoate 3430 (O–H str., phenol), 3078 (C=H str., Ar), 1733 (C=O str., ester), 1233 (C–O–C assym. str., Ar). ^1^H NMR (400 MHz, DMSO-*d*_6_): 7.69 (dd, *J* = 7.5, 1.5 Hz, 2H), 7.54 (d, *J* = 1.5 Hz, 2H), 7.11 (d, *J* = 7.4 Hz, 2H), 6.84–6.79 (m, 6H), 5.98 (ddt, *J* = 16.8, 9.9, 6.2 Hz, 2H), 5.19–5.14 (m, 3H), 5.10 (dt, *J* = 2.2, 1.0 Hz, 1H), 3.84 (d, *J* = 10.6 Hz, 12H), 3.34 (dp, *J* = 6.2, 1.0 Hz, 4H). ^13^C NMR (400 MHz, CDCl_3_): 163.8, 151.9, 151.8, 144.6, 141.1, 139.8, 138.2, 123.5, 121.5 (d, *J* = 5.5 Hz), 120.4, 119.9, 116.3, 112.1 (d, *J* = 13.9 Hz), 56.1, 40.7; m/z 314.09 [M+1]+; Anal. For C_18_H_18_O_5_: calcd: C, 68.78; H, 5.77 found: C, 68.77; H, 5.75.

**13b** 4-allyl-2-methoxyphenyl 4-hydroxybenzoate IR (KBr pellets) cm^−1^ 3450 (O–H str., phenol), 3077 (C=H str., Ar), 1708 (C=O str., ester), 1245 (C–O–C assym. str., Ar). ^1^H NMR (400 MHz, DMSO-*d*_6_): 7.94–7.82 (m, 2H), 7.12 (d, *J* = 7.5 Hz, 1H), 6.95–6.92 (m, 2H), 6.83–6.79 (m, 2H), 5.87 (tt, *J* = 13.4, 6.2 Hz, 1H), 5.14 (dddt, *J* = 21.2, 13.4, 2.1, 1.0 Hz, 2H), 3.80 (s, 3H), 3.35 (dp, *J* = 6.2, 1.0 Hz, 2H). ^13^C NMR (400 MHz, CDCl_3_): 161.7 (d, *J* = 3.2 Hz), 149.9, 140.1, 139.8, 137.2, 132.6, 120.6–120.2 (m), 117.4, 117.3, 114.6, 55.9, 40.4; m/z 284.09 [M+1]+; Anal. For C_17_H_16_O_4_: calcd: C, 71.82; H, 5.67 found: C, 71.80; H, 5.65.

**13c** 4-allyl-2-methoxyphenyl 2-hydroxybenzoate IR (KBr pellets) cm^−1^ 3444 (O–H str., phenol), 3076 (C=H str., Ar), 1747 (C=O str., ester), 1252 (C–O–C assym. str., Ar); ^1^H NMR (400 MHz, DMSO-*d*_6_): 7.99 (dd, *J* = 7.5, 1.5 Hz, 2H), 7.62 (td, *J* = 7.5, 1.5 Hz, 2H), 7.12 (d, *J* = 7.5 Hz, 2H), 7.09 (td, *J* = 7.5, 1.5 Hz, 2H), 6.91 (dd, *J* = 7.5, 1.5 Hz, 2H), 6.85–6.77 (m, 4H), 5.99 (ddt, *J* = 16.8, 9.9, 6.2 Hz, 2H), 5.19–5.11 (m, 3H), 5.07 (dt, *J* = 2.2, 1.0 Hz, 1H), 3.87 (s, 6H), 3.22 (dp, *J* = 6.2, 1.0 Hz, 4H). ^13^C NMR (400 MHz, CDCl_3_): 170.6, 160.5, 152.9, 139.8, 139.5, 138.2, 135.7, 131.6, 119.5 (d, *J* = 5.5 Hz), 119.8, 118.5, 115.3, 114.8, 113.6, 56.9, 41.4; m/z 284.1 [M+1]+; Anal. For C_17_H_16_O_4_: calcd: C, 71.82; H, 5.67 found: C, 71.81; H, 5.65.

**13d** 4-allyl-2-methoxyphenyl 2-mercaptobenzoate IR (KBr pellets) cm^−1^ 3344 (C=H str., Ar), 1717 (C=O str., ester), 1260 (C–O–C assym. str., Ar), 643 (C=S str., thiol). ^1^H NMR (400 MHz, DMSO-*d*_6_): 8.79 (dd, *J* = 7.4, 1.6 Hz, 2H), 7.56 (dd, *J* = 7.4, 1.6 Hz, 2H), 7.47 (td, *J* = 7.4, 1.6 Hz, 2H), 7.34 (td, *J* = 7.4, 1.6 Hz, 2H), 7.12 (d, *J* = 7.5 Hz, 2H), 6.90–6.78 (m, 4H), 5.99 (ddt, *J* = 16.8, 9.9, 6.2 Hz, 2H), 5.17–5.11 (m, 3H), 5.09 (dt, *J* = 2.2, 1.0 Hz, 1H), 3.86 (s, 6H), 3.24 (dp, *J* = 6.2, 1.0 Hz, 4H). ^13^C NMR (400 MHz, CDCl_3_): 165.4, 150.9, 140.8, 140.5, 137.2, 136.5, 133.1, 131.5, 130.8, 128.8, 125.4, 121.5 (d, *J* = 5.5 Hz), 117.3, 112.6, 59.9, 47.5; m/z 300.06 [M+1]+; Anal. For C_17_H_16_O_3_S: calcd: C, 67.98; H, 5.37 found: C, 67.97; H, 5.35.

**16** (Z)-4-allyl-2-methoxyphenyl 3-(4-hydroxy-3-methoxyphenyl)acrylate IR (KBr pellets) cm^−1^ 3509 (O–H str., phenol), 3075 (C=H str., Ar), 1720 (C=O str., ester), 1206 (C–O–C assym. str., Ar). ^1^H NMR (400 MHz, DMSO-*d*_6_): 7.55 (d, *J* = 1.6 Hz, 1H), 7.25–7.17 (m, 1H), 7.09 (d, *J* = 7.8 Hz, 1H), 6.99 (ddd, *J* = 7.5, 1.5, 0.6 Hz, 1H), 6.76 (tdd, *J* = 3.5, 1.5, 0.8 Hz, 2H), 6.72 (d, *J* = 7.5 Hz, 1H), 6.18 (d, *J* = 10.9 Hz, 1H), 5.94 (ddt, *J* = 16.8, 9.9, 6.2 Hz, 1H), 5.20–5.11 (m, 1H), 5.11–5.06 (m, 1H), 3.85 (d, *J* = 10.6 Hz, 6H), 3.32 (dp, *J* = 6.2, 1.0 Hz, 2H). ^13^C NMR (400 MHz, CDCl_3_): 164.4, 151.3, 149.6, 148.8, 144.8, 140.2, 139.8, 138.2, 126.2, 123.9, 121.6, 120.8, 116.3, 115.6, 115.1, 113.2, 111.8, 56.2, 55.9, 40.4; m/z 340.11 [M+1]+; Anal. For C_20_H_20_O_5_: calcd: C, 70.57; H, 5.92 found: C, 70.55; H, 5.91.

**17** (Z)-4-allyl-2-methoxyphenyl 3-phenylacrylate IR (KBr pellets) cm^−1^ 3077 (C=H str., Ar), 1700 (C=O str., ester), 1206 (C–O–C assym. str., Ar). ^1^H NMR (400 MHz, DMSO-*d*_6_): 7.61 (ddt, *J* = 6.5, 1.6, 0.7 Hz, 2H), 7.41–7.36 (m, 1H), 7.36–7.27 (m, 2H), 7.18 (dt, *J* = 10.9, 0.7 Hz, 1H), 7.10 (d, *J* = 7.8 Hz, 1H), 6.88 (tdd, *J* = 3.6, 1.5, 0.8 Hz, 2H), 6.13 (d, *J* = 10.9 Hz, 1H), 5.80 (tt, *J* = 13.4, 6.2 Hz, 1H), 5.13 (dddt, *J* = 21.2, 13.4, 2.1, 1.0 Hz, 2H), 3.78 (s, 3H), 3.29 (dp, *J* = 6.2, 1.0 Hz, 2H). ^13^C NMR (400 MHz, CDCl_3_): 159.5, 153.3, 145.2, 142.2, 141.8, 137.2, 134.6, 130.1, 128.8, 128.5, 122.6, 120.8, 116.9 (d, *J* = 13.3 Hz), 113.2, 55.9, 41.4; m/z 294.11 [M+1]+; Anal. For C_19_H_18_O_3_: calcd: C, 77.53; H, 6.16 found: C, 77.51; H, 6.14.

### Recombinant human MAO inhibition screening

To evaluate IC_50_ values for the inhibition of human MAO-A and MAO-B by newly synthesized compounds the commercially available recombinant human enzymes expressed in insect cells were used (Sigma-Aldrich). The MAO inhibition assay was based on the method described by Anderson et al., 1993 and Chimenti et al. with some modifications [27, 28]. The Amplex Red MAO Kit tyramine (Sigma-Aldrich) affords a suitable fluorimetric method to evaluate MAO enzyme action of synthetic and natural compounds. In the assay, MAO reacts with *p*-tyramine, a common substrate for both MAO isoforms, ensuing in the production of H_2_O_2_, which is identified by a fluorimetric method (λ_ex_ = 530/λ_em_ = 585 nm). Each test well contained 0.1 mL of sodium phosphate buffer (0.05 M, pH 7.4), a range of concentrations of the eugenol based derivatives or reference compounds along with recombinant hMAO-A or hMAO-B in appropriate quantity. After the preparation of this mixture the wells were subjected to incubation for 15 min at 37 °C, placed in the dark fluorimeter chamber. Once this incubation period over, the process was rendered by mixing 200 lM Amplex Red reagent (Sigma-Aldrich), 1 U/mL horseradish peroxidase (Sigma-Aldrich) and *p*-tyramine (Sigma-Aldrich). Subsequently, the formation of hydrogen peroxide exerted by MAO isoforms was quantified by using Amplex-Red reagent, a non-fluorescent probe that reacts with H_2_O_2_ in the existence of horse radish peroxidase to generate the fluorescent component resorufin. Moreover, the control analysis was calculated in the absence of compound and reference inhibitors. The probability of compounds to change the fluorescence produced in the reaction mix due to non-enzymatic inhibition was assessed by mixing titled compounds to solutions of single Amplex Red reagent in a sodium phosphate buffer. Data were processed in Microsoft Excel, to calculate IC_50_ values.

### In silico ADME prediction

The in silico ADMET evaluation of the synthesized compounds was performed by QikProp software by Schrödinger suit. Several descriptors for determination of essential pharmacological properties were calculated for instance Lipinski rule, total polar surface area, MDCK, Caco-2, no. of rotatable bonds, logPoct and logBB. Caco-2 cell and Madin–Darby canine kidney (MDCK) cell models are considered crucially unfailing in vitro model to estimate the oral drug absorption. For a CNS acting drug the logBB is an important descriptor to identify blood–brain barrier (BBB) diffusion and it is estimated as the concentration range of compound concentration in peripheral blood (C_blood_) and brain (C_brain_) [29–33]. The ADMET score calculated by QikProp showed the crucial parameters for the drug-like properties of the synthesized compounds.

### Enzyme kinetics

Lineweaver–Burk plots for the inhibition of MAO-A and MAO-B were constructed by using p-tyramine at eight different concentrations for each plot (15–250 μM). For each test inhibitor, six Lineweaver–Burk plots were constructed. Plotting of Michaelis–Menten curves was used to estimate inhibition data by global fitting of the to the Michaelis–Menten equation using the Prism 5 software package.

### Preparation of ligands

The 3D structures for all the eugenol based derivatives were designed in MDL MOL format and then processed by LigPrep module of Schrödinger. The LigPrep utilized to optimize and assign the accurate protonation pose and torsions to all ligands. Finally the stereochemical structures around 32 was done at pH state 7.0 ± 2.0 via utilizing ionizer. The desalting of ligands and optimization and tautomerization was done by utilizing OPLS 2005 force field.

### Preparation of proteins

The 3-D crystallographic protein structures for hMAO-A and hMAO-B at good resolution were taken from the Protein Data Bank with succession codes 2Z5X [34] and 2V5Z [35] respectively. Prior to molecular docking the removal of inhibitors as co-crystallized form, safinamide for 2V5Z and harmine for 2Z5X and structural water molecules was done from hMAO-A and hMAO-B proteins. The explicit active sites were precisely set at 1000 A3 standard grid box located near cofactor FAD’s N5 atom. Consequently, the protonation and charge position were allocated and minimization of energy was done via OPLS2005 force field.

### Docking methodology

Molecular docking was carried out by implementing flexible algorithm with XP approach of Glide. The grid box was generated frequently for assortment and formation of centroid of the dynamic site residues and the integral receptor protein was processed via energy minimization at RMSD rate of 0.17 Å. The side chains of B-factor in MAO protein were separated before docking experiments. All of the amino acid residues were refined and energy minimization and optimization of primary side chains. The aforementioned process exposed the correct site of ligand structure and conformation for induced fit docking. Therefore, with the XP Glide mode redocking was performed which afforded dock score for every ligand.

### Determination of DPPH scavenging activity

The antioxidant activity of the newly synthesized compound was measured on the basis of the scavenging activity of the stable 1, 1-diphenyl 2-picrylhyorazyl (DPPH) free radical The evaluation of the antioxidant activity was based on free radical scavenging effect of stable DPPH· with spectrophotometric titration [36, 37]. Solutions of synthesized compounds were set in ethanol/water (1:1) having 10 mM to 100 mM at a period of 10 mM. The 0.01% DPPH· stock solution was prepared in ethanol/water (1:1). Samples were prepared as 5 mL DPPH· solution was mixed with 5 mL l-ascorbic acid solution then shaken vigorously and kept in the dark. Corresponding blank sample were prepared and l-ascorbic acid was used as reference standard. All samples were subjected for incubation at 37 °C for an hour in the dark chamber prior to evaluate absorbance. To evaluate the absorption spectra, firstly blank spectra for ethanol/water were observed afterward the spectrophotometric titration was carried out at various concentrations of titled compounds. The absorbance was calculated at 517 nm with UV/Vis Epoch ELISA reader, and radical scavenging activity was calculated as a reduction in absorbance of DPPH·. The actual decrease in absorption induced by the test was compared with the positive controls. The IC_50_ values were calculated use the dose inhibition curve in linear range by plotting the extract concentration versus the corresponding scavenging effect through following equation [(A_DR_ − A_PR_)/A_O_] × 100. Where A_DR_ is the absorbance prior to reaction and A_PR_ is the absorbance after reaction with DPPH·.

### Hydrogen peroxide scavenging (H_2_O_2_) assay

Hydrogen peroxide generally turns into water and oxygen and produces highly toxic hydroxyl radical that renders the damage to DNA and peroxidation of lipid. H_2_O_2_ scavenging ability of eugenol based derivatives was evaluated by method described by Nakano and colleagues with some alteration [38, 39]. The hydrogen peroxide solution (40 mM) was prepared in phosphate buffer (40 mM pH 7.4) and addition of several ranges of synthesized derivatives (05–80 μg/mL) was done into H_2_O_2_ solution (2 mL). The determination of H_2_O_2_ concentration was done by UV/Vis Epoch ELISA reader at 230 nm upon incubation of 10 min. Solution of phosphate buffer without H_2_O_2_ was noted as blank reading. Antioxidant potential of synthesized compounds is shown in Table 3. The hydrogen peroxide percentage inhibition was estimated through the formula [(A_H _− A_D_)/A_O_] × 100, where A_H_ is the absorbance of the control and A_D_ is the absorbance of compounds/reference taken as 05–80 μg/mL l-ascorbic acid.

### Nitric oxide scavenging activity

The generation of nitric oxide (NO·) on biological tissues is mediated by particular nitric oxide synthases enzyme that control the catabolism of the arginine to citrulline via creation of NO through a five electron oxidative reaction. At the physiological pH (7.2) in aqueous solution sodium nitroprusside decompose to produce NO·. Finally, NO· reacts with oxygen at underaerobic conditions and produces stable products (nitrate and nitrite), this amount can be determined by means of Griess reagent. The procedure for the nitric oxide scavenging activity was adopted from reports of Alam et al. [40].

### Ferric reducing-antioxidant power (FRAP) assay

The capacity of antioxidants to decrease ferric iron is measured by this method. The reaction of 2,3,5-triphenyl-1,3,4-triaza-2-azoniacyclopenta-1,4-diene chloride (TPTZ) and complex ferric iron yields ferrous form at low pH. The monitoring of the above reduction reaction at 593 nm under ELISA reader reveals the antioxidant potential. This method was adopted from the reports of Benzie et al. [41].

## Conclusion

All of the research findings facilitated to conclude the structural specificity rationale for the hMAO-B and hMAO-A of this new series of natural eugenol based inhibitors. The results of the study improved our confidence in our scheme and encouraged us to carry on our study to design more persuasive and selective inhibitors. The considerable hMAO-A inhibitory potential of compounds **5b** with IC_50_ value (5.989 ± 0.007 µM) might be due to presence of 3,4,5-trihydroxybenzoy-thiosemicarbazide unit within the **5b** structure. Moreover the compound **16** exhibiting 4-hydroxy-3-methoxyphenyl-acrylate on 4-allyl-2-methoxyphenyl moiety has proven the importance of esteric linkage for h-MAOA active site. In case of compound **13b** the 4-hydroxybenzoate linkage with eugenol enhanced the hMAO-B inhibition. I was observed that methoxy group slightly reduced the hMAO-B inhibitory efficacy as compared with **13a** (having single hydroxyl group at aromatic ring). Together with significant MAO inhibitory activity of abovementioned derivatives the future trend is directed towards pre-clinical studies. The reduction of physiological antioxidants in the CNS causes excessive oxidative stress by overproduction of MAO in brain and is concerned with neurodegenerative disorders. We synthesized the natural eugenol based compounds which exhibits considerable MAO inhibitory potential along with antioxidant activity that might probably pay for foremost advantages to explore the fundamental neurological pathology.
